# Edge Artificial Intelligence Device in Real-Time Endoscopy for the Classification of Colonic Neoplasms

**DOI:** 10.3390/diagnostics15121478

**Published:** 2025-06-10

**Authors:** Eun Jeong Gong, Chang Seok Bang

**Affiliations:** 1 Department of Internal Medicine, Hallym University College of Medicine, Chuncheon 24253, Republic of Korea; gong-eun@hanmail.net; 2 Institute for Liver and Digestive Diseases, Hallym University, Chuncheon 24253, Republic of Korea; 3 Institute of New Frontier Research, Hallym University College of Medicine, Chuncheon 24253, Republic of Korea; 4 Division of Big Data and Artificial Intelligence, Chuncheon Sacred Heart Hospital, Chuncheon 24253, Republic of Korea

**Keywords:** colonoscopy, deep learning, endoscopy, colonic neoplasms

## Abstract

**Objective:** Although prior research developed an artificial intelligence (AI)-based classification system predicting colorectal lesion histology, the heavy computational demands limited its practical application. Recent advancements in medical AI emphasize decentralized architectures using edge computing devices, enhancing accessibility and real-time performance. This study aims to construct and evaluate a deep learning-based colonoscopy image classification model for automatic histologic categorization for real-time use on edge computing hardware. **Design:** We retrospectively collected 2418 colonoscopic images, subsequently dividing them into training, validation, and internal test datasets at a ratio of 8:1:1. Primary evaluation metrics included (1) classification accuracy across four histologic categories (advanced colorectal cancer, early cancer/high-grade dysplasia, tubular adenoma, and nonneoplasm) and (2) binary classification accuracy differentiating neoplastic from nonneoplastic lesions. Additionally, an external test was conducted using an independent dataset of 269 colonoscopic images. **Results:** For the internal-test dataset, the model achieved an accuracy of 83.5% (95% confidence interval: 78.8–88.2%) for the four-category classification. In binary classification (neoplasm vs. nonneoplasm), accuracy improved significantly to 94.6% (91.8–97.4%). The external test demonstrated an accuracy of 82.9% (78.4–87.4%) in the four-category task and a notably higher accuracy of 95.5% (93.0–98.0%) for binary classification. The inference speed of lesion classification was notably rapid, ranging from 2–3 ms/frame in GPU mode to 5–6 ms/frame in CPU mode. During real-time colonoscopy examinations, expert endoscopists reported no noticeable latency or interference from AI model integration. **Conclusions:** This study successfully demonstrates the feasibility of a deep learning-powered colonoscopy image classification system designed for the rapid, real-time histologic categorization of colorectal lesions on edge computing platforms. This study highlights how nature-inspired frameworks can improve the diagnostic capacities of medical AI systems by aligning technological improvements with biomimetic concepts.

## 1. Introduction

Colorectal cancer remains a significant public health challenge globally due to its high incidence and mortality rates [1]. Early detection and accurate histological classification through colonoscopy are critical for improving patient outcomes and guiding effective treatment strategies [2]. Colonoscopy allows for direct visual assessment and removal of colorectal lesions, significantly reducing the risk of progression to invasive cancer [3].

Despite these advantages, conventional colonoscopic evaluation relies heavily on endoscopists’ subjective judgment and experience, resulting in variability in diagnosis accuracy [4]. Histological confirmation via biopsy, while accurate, introduces additional delays, costs, and procedural complexities [5]. These limitations underscore the need for enhanced, reliable, and immediate diagnostic techniques.

Artificial intelligence (AI), particularly deep learning algorithms, has demonstrated considerable promise in improving diagnostic accuracy across various medical imaging domains, including endoscopy [6,7]. Previous studies utilizing AI for colorectal polyp classification have shown potential for assisting endoscopists in achieving more consistent and precise diagnoses [8]. However, most AI systems require powerful computing resources, typically relying on centralized cloud or high-end computing infrastructures, posing challenges such as latency issues, data privacy concerns, and dependence on continuous internet connectivity [9].

Recent advancements in edge computing technology offer promising solutions to these practical barriers. Edge computing facilitates the execution of AI models locally on medical devices, significantly reducing latency, enhancing data security, and minimizing reliance on internet connectivity. These features make edge computing particularly suitable for real-time clinical decision-making environments, such as endoscopic examinations [10].

This study aims to leverage these advancements to develop and validate a novel optimized deep-learning classification model specifically designed for deployment on an edge computing platform. The primary objective is to automatically and accurately classify colorectal lesions in real time during endoscopy, distinguishing between advanced colorectal cancer, early cancers/high-grade dysplasia, tubular adenomas, and nonneoplastic lesions, as well as differentiating broadly between neoplastic and nonneoplastic lesions. Through the integration of biomimetic principles with technological advancements, this study seeks to demonstrate how nature-inspired frameworks might enhance the diagnostic capabilities of medical AI systems.

In colorectal neoplasia, standardized classification systems provide a framework to stratify lesions by malignant potential. The revised Vienna classification delineates five categories of gastrointestinal epithelial neoplasia: Category 1 (negative for neoplasia/dysplasia), Category 2 (indefinite for neoplasia/dysplasia), Category 3 (mucosal low-grade neoplasia, e.g., tubular adenoma), Category 4 (mucosal high-grade neoplasia, such as high-grade dysplasia or intramucosal carcinoma), and Category 5 (invasive neoplasia with submucosal or deeper invasion). The World Health Organization (WHO) histological classification similarly distinguishes benign colorectal adenomas from malignant adenocarcinomas, using the presence of submucosal invasion as the key criterion for malignancy—consistent with the Vienna system’s division between non-invasive (categories 1–4) and invasive (category 5) lesions [11]. In this study, our AI model was trained to categorize lesions in analogous terms—nonneoplastic, tubular adenoma, early cancer/high-grade dysplasia, and advanced cancer—mirroring these classification schemes and emphasizing the overarching dichotomy between neoplastic and nonneoplastic findings.

We hypothesize that the integration of AI into an edge computing platform will significantly improve real-time diagnostic accuracy, reduce unnecessary biopsies, enhance early colorectal cancer detection rates, and standardize diagnostic practices among endoscopists. Ultimately, this research aims to validate an effective, clinically deployable AI solution that can seamlessly integrate into routine colorectal cancer screening and surveillance programs, improving both clinical efficiency and patient outcomes.

## 2. Materials and Methods

**Figure 1:**
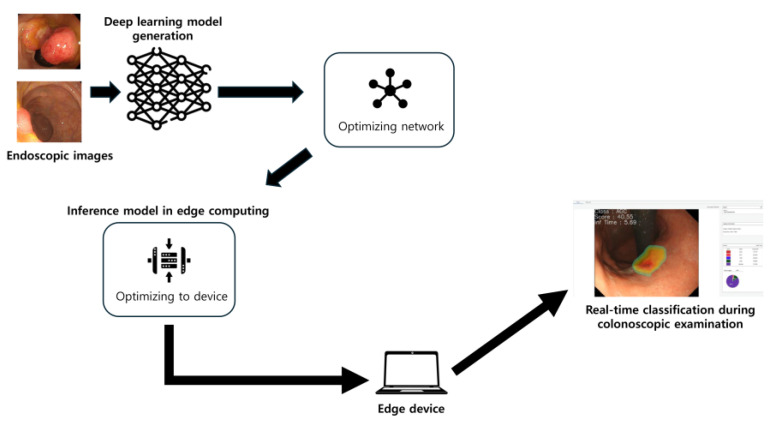
Overall design of this study.

The overall study design is illustrated in Figure 1. This study extends previous research, with the key difference being the establishment of the AI model and an edge device [12,13,14].

### 2.1. Data Acquisition

This study utilized a carefully selected subset of colonoscopy images based on the previous finding that optimal model performance was achieved with a total of 2418 images. These images were originally gathered retrospectively from patients who underwent colonoscopic examinations and subsequent polypectomies between 2008 and 2017 at three affiliated hospitals (Chuncheon Sacred Heart Hospital, Dongtan Sacred Heart Hospital, and Hallym University Sacred Heart Hospital). All included images had corresponding histopathological confirmations and were saved in JPEG format at a resolution of at least 640 × 480 pixels [12].

The final dataset of 2418 images was specifically selected based on previous research [13], which demonstrated that this specific dataset volume and distribution provided the highest accuracy for colorectal polyp classification. The dataset included representative proportions of the four major colorectal lesion categories: advanced colorectal cancer, early colorectal cancers/high-grade dysplasia, tubular adenomas, and nonneoplastic lesions. GIF-Q260, H260, or H290 endoscopes (Olympus Optical Co., Ltd., Tokyo, Japan) were used for all internal tests and training exams, together with an endoscopic video imaging system (Evis Lucera CV-260 SL or Elite CV-290; Olympus Optical Co., Ltd., Tokyo, Japan). The detailed number of each category in the training dataset is presented in Table 1.

An additional independent external-test dataset comprising 269 images was obtained from colonoscopy procedures conducted at several hospitals not involved in the original data collection (including Kangdong Sacred Heart Hospital, Inje University Ilsan Paik Hospital, and Gangneung Asan Hospital) to assess the model’s generalizability to unseen data. This external dataset allowed for rigorous validation of the model’s predictive performance in real-world clinical scenarios. GIF-Q260, H260, or H290 endoscopes (Olympus Optical Co., Ltd., Tokyo, Japan) were used for all external tests and training exams, together with an endoscopic video imaging system (Evis Lucera CV-260 SL or Elite CV-290; Olympus Optical Co., Ltd., Tokyo, Japan). The detailed number of each category in the external-test dataset is presented in Table 1.

### 2.2. Image Labeling

All images were annotated according to the histopathological diagnosis of the lesion, which served as the ground truth for classification. Each image was assigned to one of four diagnostic categories based on pathology results, defined as follows: Advanced Colorectal Cancer (ACC): Invasive adenocarcinoma involving the muscularis propria or beyond (pathologic stage T2 or higher). Early Colorectal Cancer/High-Grade Dysplasia (ECC/HGD): Early-stage carcinoma confined to the mucosa or submucosa (pathologic T1 or carcinoma in situ), including adenomatous polyps with high-grade dysplasia. Tubular Adenoma (TA): Benign tubular adenomas, including those with low-grade dysplasia or no dysplasia. Nonneoplastic Lesion: Benign, nonneoplastic findings such as hyperplastic polyps, inflammatory polyps, lymphoid polyps, lipomas, or other similar lesions.

Each image was labeled exclusively with one of these four classes, with no overlap between categories. These class definitions reflect the lesion’s pathology and clinical significance, and they formed the four target output classes for the classification model. All images were cross-checked by two expert endoscopists (C.S.B. and E.J.G.) to minimize inter-observer variability and guarantee appropriate categorization.

### 2.3. Model Development

Model development was carried out using a no-code deep learning platform (Neuro-X, version 3.3.1; Neurocle Inc., Seoul, Republic of Korea). This platform provides an environment for building and training deep neural networks for image analysis without manual coding. It automatically generates an optimized convolutional neural network model for classification by analyzing the input dataset and performing automated hyperparameter tuning. In this way, a consistent training pipeline was applied across all experiments, and the model architecture selection and optimization were handled by the platform’s built-in algorithms. All model training was executed on premises using Neuro-X’s default training pipeline, which includes automated image preprocessing. Specifically, all input images were resized to a uniform resolution of 512 × 480 pixels before being fed into the model. The platform then randomly divided each dataset into an 8:1:1 ratio for the training, validation, and internal-test datasets. This splitting procedure was applied consistently for all the training dataset variants described above. Model training was performed using Neuro-X’s automated hyperparameter optimization mode, which iteratively adjusted network parameters (such as learning rate, network depth, and other hyperparameters) to maximize performance on the internal-validation subset. Model training was accelerated using a high-performance computing system equipped with four NVIDIA RTX 2080 Ti GPUs, dual Intel Xeon processors, and 256 GB of RAM. This on-premises hardware setup enabled efficient processing of the deep learning training tasks within the Neuro-X environment, aligning with an edge-computing approach to model development (i.e., all computations were done locally rather than in the cloud) [14]. The final trained model used for evaluation was selected based on the highest classification accuracy achieved on the internal-validation data.

### 2.4. Establishment of Edge Device

In this study, the deep learning model was deployed on a custom edge computing device configured with a high-end laptop processor (Intel i9-13980HX) and GPU (NVIDIA RTX 4090) to enable GPU-accelerated, real-time inference at the point of care. All inference software was implemented in C++ (version 23), chosen for its efficient low-level hardware control, and bundled with a graphical user interface that allows clinicians to execute the model and visualize results through simple button clicks with no coding required. The interface supports three input modalities—live endoscopic video streams, still images, and recorded video files—accepting standard image formats (e.g., JPEG, PNG, BMP). During operation, it overlays the model’s classification results on the incoming endoscopic image and provides a summary panel of inference metrics (such as processing speed per frame and classification probabilities) in real time. To achieve high throughput and minimal latency, the inference engine was optimized using several techniques, including (1) parallel computing across the CPU and GPU to maximize the utilization of computing resources, (2) automatic mixed-precision computation (FP32 → FP16 via AMP) to accelerate math operations while preserving accuracy, (3) layer and tensor fusion to reduce data transfer and memory overhead between network layers, (4) GPU kernel auto-tuning tailored to the RTX 4090 for optimal low-level performance, and (5) dynamic tensor memory management for efficient reuse of GPU memory. Additionally, the system integrates the OpenCV library for fast image capture and preprocessing and employs OpenMP for multi-threaded CPU parallelism, further streamlining real-time data handling. This tailored hardware–software implementation enabled real-time model inference during live endoscopy with negligible latency—in testing, the device processed frames in roughly 2–3 milliseconds on the GPU (5–6 ms on the CPU for 640 × 380 images) and introduced no perceptible delay in lesion classification as reported by the endoscopists [10].

### 2.5. Evaluation Metrics

The performance of the classification model was evaluated using several standard metrics. The primary outcome measure was classification accuracy, calculated as the proportion of correctly classified images out of all images in the test set. In addition, precision (positive predictive value) and recall (sensitivity) for each class were calculated along with the overall F1-score to summarize the balance between precision and recall. Precision was defined as the fraction of cases predicted as a given class that were actually that class (i.e., Precision = true positive/true positive + false positive for each class). Recall was defined as the fraction of true cases of a class that were correctly identified by the model (Recall = true positive/true positive + false negative). The F1-score, which is the harmonic mean of precision and recall, was computed as 2 precision * recall/precision + recall. These metrics were first evaluated on the internal-validation set during model training and selection. After the model was finalized, the same metrics were reported on the external test set to assess the model’s performance and generalization on independent data [10,12,13,14].

### 2.6. Ethics Approval

This study was conducted as a retrospective analysis of de-identified endoscopy images and was approved by the Institutional Review Board of Chuncheon Sacred Heart Hospital. The requirement for informed consent was waived by the IRB due to the use of pre-existing, anonymized clinical data.

### 2.7. Analysis of Attention Maps for Explainability

Gradient-weighted class activation mapping (Grad-CAM) was integrated into the deep learning model to generate attention maps highlighting the regions most influential for each prediction. These attention maps were overlaid on the endoscopic image output and displayed in real time on the edge device during inference, thereby providing the endoscopist with immediate visual insight into the AI’s decision-making process. The attention maps were not formally quantified in this study; instead, a post hoc qualitative review of the external-test cases was conducted by two expert endoscopists (C.S.B. and E.J.G.) to assess whether the AI system’s focus corresponded to clinically relevant features of the lesions. Representative examples of both correct and incorrect model classifications, along with their corresponding attention maps, were selected for presentation in the Results section to illustrate instances where the model’s focus aligned appropriately with pathology and instances where it did not, underscoring the strengths and limitations of the attention-based explainability approach [10,12,13,14].

## 3. Results

### 3.1. Features of the Dataset

In total, 2418 endoscopic images were collected retrospectively and split into training, validation, and internal-test datasets at random in an 8:1:1 ratio. ‘ACC’ and ‘ECC/HGD’ accounted for the largest percentage of the total images [33.3% (806/2418)], followed by ‘TA’ and ‘Nonneoplasm’ [16.7% (403/2417)].

For the external-test set, 269 unique images that were not in the training, validation, or internal-test datasets were collected. Following “nonneoplasm” [56.1%, (151/269)] and “TA” [34.2%, (92/269)], the proportion of “ECC/HGD” was the greatest [5.9%, (16/269)]. The quantity and distribution of each category in the dataset are shown in Table 1.

### 3.2. Hyperparameters for Training

The AI model was established using the on-premise software’s proprietary neural network structure, Momentum optimizer, a batch size of 72, an epoch count of 100, a patience value of 30, and the cosine learning rate decay method (decay ends after 2400 steps, initial learning rate 0.002). Categorical cross-entropy was used as the loss function. At epoch 58/89, the training loss was 0.0548, and the validation loss was 0.0369.

### 3.3. Performance of Internal Test

**Figure 2:**
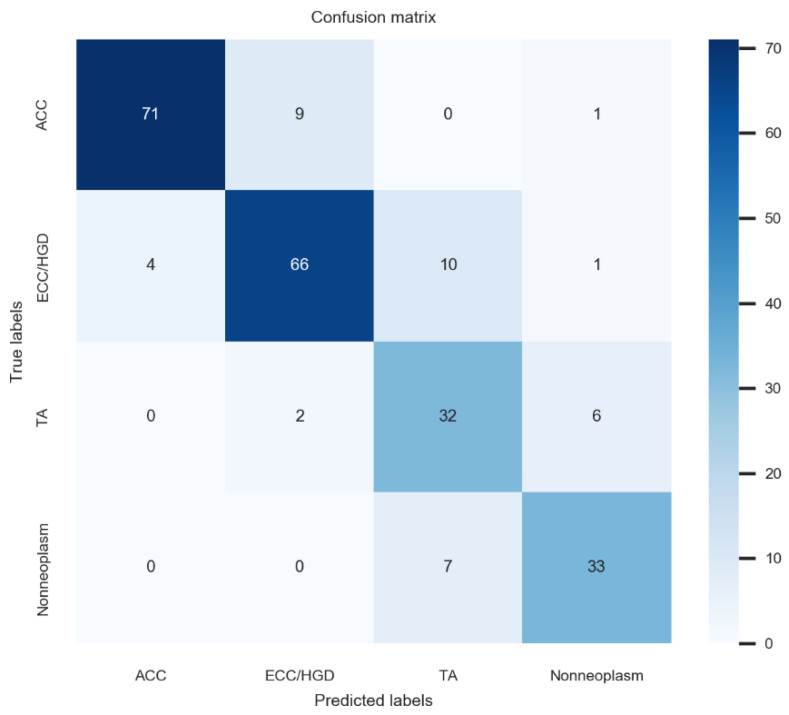
Four-class confusion matrix for the internal test. ACC, advanced colorectal cancer; ECC/HGD, early colorectal cancer/high-grade dysplasia; TA, tubular adenoma.

In the internal test, the established model’s accuracy was 83.5% (95% confidence interval: 78.8–88.2%) for the four-category classification, with a precision of 81.5% (76.6–96.4%), a recall of 82.9% (78.2–87.6%), and an F1-score of 82.2% (77.4–87.0%). In binary classification (neoplasm vs. nonneoplasm), accuracy improved significantly to 94.6% (91.8–97.4%), with a precision of 88.6% (84.6–92.6%), a recall of 93.8% (90.8–96.8%), and an F1-score of 91.1% (87.5–94.7%). The confusion matrix for four-class internal-test performance is shown in Figure 2. Most misclassifications occurred between ECC/HGD and TA, reflecting the difficulty in distinguishing these morphologically similar lesions. Notably, the model correctly identified 93.8% of the neoplastic lesions, and the accuracy for advanced cancer was particularly high.

### 3.4. Performance of External Test

**Figure 3:**
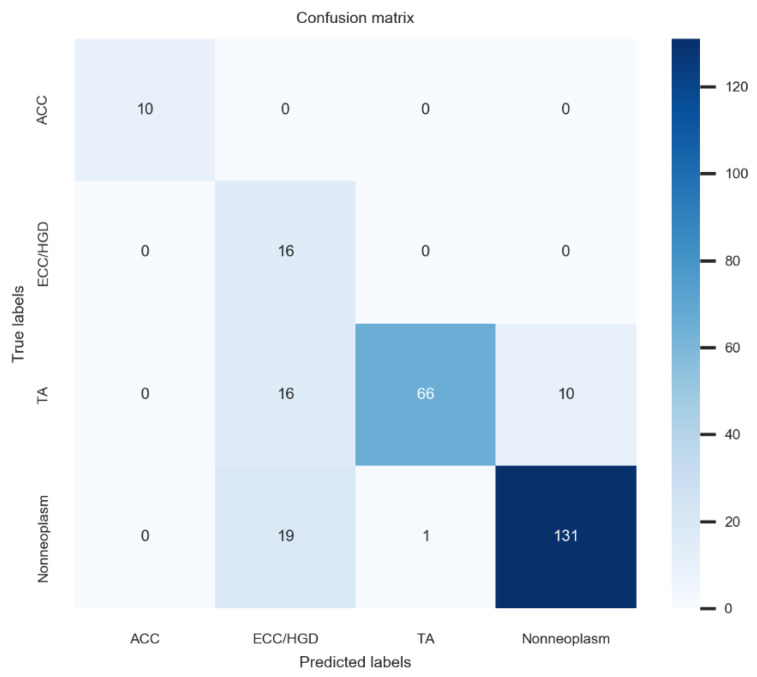
Four-class confusion matrix for the external test. ACC, advanced colorectal cancer; ECC/HGD, early colorectal cancer/high-grade dysplasia; TA, tubular adenoma.

The external test demonstrated an accuracy of 82.9% (95% confidence interval: 78.4–87.4%) in the four-category task and a notably higher accuracy of 95.5% (93.0–98.0%) for binary classification. Figure 3 demonstrates the confusion matrix for external-test performance. The model demonstrated similarly robust performance, though minor confusion persisted between TA and nonneoplastic lesions—highlighting areas for potential model refinement. The inference speed for lesion classification of the established model was 2–3 ms in GPU mode and 5–6 ms in CPU mode (employing a 640 × 380 image at a batch size of 1 on hardware comprising an i9-13980HX CPU and an RTX 4080 GPU). The expert endoscopists did not experience delays in lesion classification time or feel that the deep learning model interfered with their exams.

### 3.5. Attention Map Analysis

**Figure 4:**
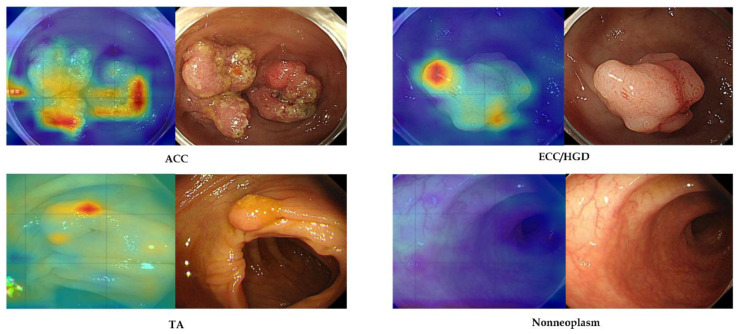
Representative images of attention map analysis. ACC, advanced colorectal cancer; ECC/HGD, early colorectal cancer/high-grade dysplasia; TA, tubular adenoma.

Figure 4 shows representative cases that were correctly identified as regions of interest by an established AI model. The right panel shows each neoplastic or nonneoplastic lesion, and the region of interest is well matched with the AI model’s focus.

## 4. Discussion

In this study, we deliberately implemented our colorectal lesion classifier on an accessible edge computing device to assess real-world clinical feasibility. Unlike purely engineering-driven projects, our focus was pragmatic—we wanted to validate that practicing clinicians could use AI during colonoscopy in real time without specialized hardware or cloud support. Edge computing enables data processing at the point of care, avoiding the latency and connectivity issues of remote servers [15]. Indeed, by running locally, our system avoids dependence on internet connections and protects patient data privacy, an important advantage in healthcare settings. The result is an AI tool that fits smoothly into the endoscopy suite. We achieved an inference speed of under 5 milliseconds per image, meaning that the model’s predictions were essentially instantaneous during live endoscopic viewing. Notably, the endoscopists reported no perceptible delay or disruption from the AI device while performing the procedures. This confirms that real-time deployment is not only technically possible but also clinically practical using modest, readily available hardware. The team’s experience suggests that a clinician-led approach to integrating AI—emphasizing usability over raw engineering optimizations—can successfully bring cutting-edge models to the bedside. We maintain an optimistic outlook that AI can be seamlessly embedded into colonoscopy workflows here and now, acting as a real-time assistant without hindering the procedure.

Our deep learning model demonstrated strong classification performance on both internal-test and external-test data. The accuracy achieved is on par with prior colonoscopy AI systems that were developed on more powerful computing platforms [14]. This high performance was maintained despite using a compact edge device, underscoring that excellent accuracy is attainable without cloud-scale resources. In terms of speed, the model’s ultra-fast inference (on the order of only a few milliseconds) is a key strength. Such speed ensures truly real-time operation—a critical requirement for AI assistance during live colonoscopies—with no noticeable lag in producing results [10]. Equally important is the model’s explainability. We incorporated Grad-CAM visual attention maps to accompany each prediction, highlighting the image regions most influential to the model’s decision [16]. In practice, these heatmaps tended to coincide with the same areas that an experienced endoscopist would examine when evaluating a lesion. This alignment provides an intuitive validation of the AI’s reasoning and can help build end-user trust. Explainable AI outputs make it easier for clinicians to understand and verify the model’s suggestions, addressing the “black box” concern often cited with deep learning. By offering a visual rationale for its classifications, the system functions not just as a predictive tool but also as a teaching aid or second pair of eyes, focusing attention on key mucosal features. Early adoption studies in endoscopy emphasize that such transparency is vital for clinician acceptance of AI. Another notable strength of our model is its generalizability. We validated the classifier on external datasets from different sources, and it retained excellent performance across these independent test sets. In other words, the AI did not overly “memorize” quirks of the development dataset, but rather learned features of colorectal lesions that are broadly applicable. This robustness against dataset shifts is encouraging, given that many AI models trained on single-center data experience accuracy degradation when applied to new hospitals or imaging conditions. Our results suggest that the model captures fundamental visual patterns of neoplasia that generalize well, which is essential for any system intended for wide clinical use. Taken together, the combination of high accuracy, real-time speed, explainable output, and cross-site generalizability positions our edge-deployed model as a compelling tool for AI-assisted colonoscopy.

In resource-constrained settings, model compression techniques can substantially reduce the computational and memory burden of deep learning models. Pruning and quantization are particularly promising. Applying such strategies to our colorectal lesion classifier would enable deployment on lower-cost edge hardware. By leveraging pruning and quantization, we can deploy our model on less powerful devices without significant performance sacrifice. These optimizations would maintain near-original accuracy and real-time inference speeds, even on hardware lacking a high-end GPU. In short, model compression broadens the accessibility of our AI system, enabling it to run effectively in lower-resource environments [17,18].

To our knowledge, this study is among the first to successfully implement real-time colorectal lesion classification on an edge device in a clinical context. Most prior research in endoscopic AI involved algorithms running on standard workstations or cloud servers, rather than on portable hardware at the bedside. Recently, our group and others have started to explore edge AI in endoscopy. Our work extends this paradigm to colonoscopy, showing that even complex tasks like multi-category colorectal lesion classification can be handled locally in real time. On the commercial front, an AI-assisted polyp detection device has already received FDA approval and is in clinical use, providing real-time computer vision support during colonoscopies. That system, and others like it, have proven that AI can increase polyp detection rates in practice—for example, studies report significant improvements in adenoma detection rates when using real-time AI as a “second observer” during colonoscopy [19]. Our study differs by targeting lesion classification (CADx) rather than detection (CADe). While CADe systems draw attention to polyps, they do not determine lesion histology or malignancy risk on the fly. There is a parallel line of research on AI-based polyp characterization. Pioneering studies by Mori et al. and others have shown that deep learning can differentiate adenomatous from hyperplastic polyps in real time using advanced imaging, approaching the performance needed for an optical biopsy strategy [6]. In fact, when clinicians use AI assistance for polyp characterization, some prospective studies have achieved >90% negative predictive value for ruling out adenomas in diminutive polyps—a threshold recommended for leaving such polyps in place [20,21]. These efforts underscore that AI has the potential not only to find lesions but also to guide management decisions during procedures. Our classifier contributes to this growing field of CADx by providing highly accurate lesion diagnoses instantaneously, which could aid endoscopists in making on-the-spot decisions about biopsies or resections. Moreover, unlike most prior CADx studies, we emphasize deployment on a cost-effective edge platform. Other researchers have experimented with lightweight models for classifying endoscopic images on embedded hardware or with fast object detectors like YOLO to flag polyps at high frame rates [22]. These reports demonstrated the technical possibility of real-time endoscopy AI. Our work advances beyond technical feasibility, offering a thorough validation of an edge AI system in a clinical-like setting with clinicians in the loop. In summary, compared to prior studies, we combine several desirable attributes—real-time operation, on-device processing, high-accuracy classification, and explainability—into one integrated system. This represents a step toward making AI a practical, everyday companion in endoscopic practice, building on the successes of earlier detection-focused systems and moving into the realm of comprehensive diagnostic support.

We acknowledge several limitations of our study, which also point to directions for future research. First, the training and internal-test data were relatively limited in size and originated from a single center. Although we did include an external test to examine generalizability, the model might still be biased by the specific imaging systems and patient population at our institution. AI models in endoscopy are known to suffer when confronted with data that differ from their training set (for example, variations in endoscope brand, bowel preparation quality, or lesion prevalence). A larger, multi-center dataset would bolster confidence that the model generalizes to diverse settings. Second, our evaluation was performed on retrospectively collected images, rather than in a prospective live clinical trial. While we simulated real-time use and even recorded demonstration videos, we did not formally measure the model’s impact on live procedure outcomes. This means we have yet to prove that using the AI assistant during actual colonoscopies will measurably improve performance metrics like adenoma detection rate or accuracy of optical diagnoses. Notably, the field of AI polyp characterization still lacks randomized trials to confirm the outcome benefits. Thus, a logical next step is a prospective study where endoscopists perform colonoscopies with the AI device in situ, to assess improvements in polyp detection, characterization accuracy, procedure time, and other clinically relevant endpoints. Third, although we used an edge computing device, it was a fairly high-performance unit with GPU acceleration. Less resourceful clinics might not have the exact device we used. The model’s current form relies on that hardware to achieve its sub-5 ms inference time; deploying it on a significantly weaker processor could affect performance. This hardware dependency is a concern for widespread adoption. Fortunately, there are engineering strategies (model pruning, quantization, distillation, etc.) that could reduce the computational load of the model. Future work will involve optimizing the model so that even lower-cost or older devices can run it effectively, widening the accessibility of this technology. Fourth, our current explainability analysis (Grad-CAM heatmaps) is qualitative and post hoc. Incorporating formal interpretability metrics would provide a more rigorous evaluation of our model’s attention, as there is indeed a recognized need for quantitative explainability assessment in AI research. In future work, we plan to integrate several quantitative interpretability metrics to validate the alignment between the model’s focus and the clinically relevant lesion regions using such metrics as intersection over union (IoU), pointing game accuracy (hit rate), or deletion/insertion curves. Fifth, while the current study focused on technical validation using retrospective image datasets, the ultimate value of such a system lies in its capacity to improve clinical outcomes. Specifically, by providing real-time histologic predictions, our model could play a key role in reducing unnecessary biopsies, improving adenoma detection and characterization, or supporting decision-making in a low-resource setting. Our future work will include a prospective study to evaluate how the use of this AI system influences key procedural metrics such as adenoma detection rate, biopsy rates, resection decisions, and diagnostic accuracy during live colonoscopy. Finally, we should consider the user interface and workflow integration aspects. In our experiments, the Grad-CAM heatmaps were displayed on the image. There is a possibility that a continuously updating heatmap could distract or obscure the endoscopist’s view if not designed carefully. We plan to refine how the AI feedback is presented—for example, by using subtle bounding boxes or translucent overlays or by triggering the display only when the model is highly confident—to ensure the AI advice remains helpful and not intrusive. We are also interested in incorporating a form of online learning or periodic retraining so the system can improve as it encounters more data in clinical use. In summary, none of these limitations undermine the core findings of our study, but they highlight important avenues to make the system even more robust and useful. Ongoing and future efforts will focus on (1) multi-center validation, testing the model on colonoscopy data from multiple hospitals and endoscope manufacturers; (2) prospective clinical trials to quantify the real-world impact on lesion detection rates, pathology concordance, and patient outcomes; (3) model and hardware optimization to maintain real-time performance on a wider range of edge devices, potentially even without a dedicated GPU; and (4) enhancing explainability and usability by improving the visualization of AI results and enabling the model to continually learn from new cases. By addressing these points, we aim to transition our system from a promising prototype to a clinically hardened tool.

In conclusion, this study successfully demonstrates the feasibility of a deep learning-powered colonoscopy image classification system designed for the rapid, real-time histologic categorization of colorectal lesions on edge computing platforms.

## Figures and Tables

**Table 1:** Data distribution for the establishment of a computer-aided classification model.

	Whole Dataset	Training Dataset	Validation Dataset	Internal-Test Dataset	External-Test Dataset
Overall	2418	1934	242	242	269
ACC	806 (33.3%)	644 (33.3%)	81 (33.5%)	81 (33.5%)	10 (3.7%)
ECC/HGD	806 (33.3%)	644 (33.3%)	81 (33.5%)	81 (33.5%)	16 (5.9%)
TA	403 (16.7%)	323 (16.7%)	40 (16.5%)	40 (16.5%)	92 (34.2%)
Nonneoplasm	403 (16.7%)	323 (16.7%)	40 (16.5%)	40 (16.5%)	151 (56.1%)

ACC, advanced colorectal cancer; ECC/HGD, early colorectal cancer/high-grade dysplasia; TA, tubular adenoma.

## Data Availability

All the data are accessible and available upon request from the corresponding author. All investigators had access to the final dataset.

## Abbreviations

AI	artificial intelligence
